# Action Recognition in a Crowded Environment

**DOI:** 10.1177/2041669517743521

**Published:** 2017-12-21

**Authors:** Laura Fademrecht, Judith Nieuwenhuis, Isabelle Bülthoff, Nick Barraclough, Stephan de la Rosa

**Affiliations:** Department of Human Perception, Cognition and Action, 28328 Max PlanckInstitute for Biological Cybernetics, Tübingen, Baden-Württemberg, Germany; Department of Human Perception, Cognition and Action, Max Planck Institute for Biological Cybernetics, Tübingen, Baden-Württemberg, Germany; Department of Psychology, University of York, UK; Department of Human Perception, Cognition and Action, 28328 Max Planck Institute for Biological Cybernetics, Tübingen, Baden-Württemberg, Germany

**Keywords:** action recognition, adaptation, crowding, eccentricity, peripheral vision

## Abstract

So far, action recognition has been mainly examined with small point-light human stimuli presented alone within a narrow central area of the observer’s visual field. Yet, we need to recognize the actions of life-size humans viewed alone or surrounded by bystanders, whether they are seen in central or peripheral vision. Here, we examined the mechanisms in central vision and far periphery (40° eccentricity) involved in the recognition of the actions of a life-size actor (target) and their sensitivity to the presence of a crowd surrounding the target. In Experiment 1, we used an action adaptation paradigm to probe whether static or idly moving crowds might interfere with the recognition of a target’s action (hug or clap). We found that this type of crowds whose movements were dissimilar to the target action hardly affected action recognition in central and peripheral vision. In Experiment 2, we examined whether crowd actions that were more similar to the target actions affected action recognition. Indeed, the presence of that crowd diminished adaptation aftereffects in central vision as wells as in the periphery. We replicated Experiment 2 using a recognition task instead of an adaptation paradigm. With this task, we found evidence of decreased action recognition accuracy, but this was significant in peripheral vision only. Our results suggest that the presence of a crowd carrying out actions similar to that of the target affects its recognition. We outline how these results can be understood in terms of high-level crowding effects that operate on action-sensitive perceptual channels.

## Introduction

In recent years, an increasing number of researchers point to the necessity that a full understanding of human social behaviour requires probing social cognitive processes, such as action recognition, under more natural experimental conditions (De Jaegher, Di Paolo, & Gallagher, 2010; Schilbach et al., 2013). Yet, surprisingly little work has been done in this regard. For example, nonverbal social interactions in real life often require humans to recognize different actions that appear in the visual periphery. Take for example a situation where you are out with a group of friends and everyone is chatting with each other. Whilst you are talking and directing your gaze to one of your friends, your peripheral vision might help you to notice another friend who is frequently checking his watch. Here, actions viewed in the periphery might serve to direct your attention towards socially relevant gaze locations. Yet, whether we are able to identify actions in the visual periphery under such conditions is so far poorly understood.

There is some evidence that probing action recognition under more naturalistic conditions provides results that differ from those obtained with standard psychophysical setups. For example, Keefe, Wincenciak, Jellema, Ward, and Barraclough (2016) used life-size photo-realistic actors presented three-dimensionally and their results indicate that complex judgments about the actors (the actor’s expectation about the weight of a box to be lifted) are different depending on whether participant view the stimuli on large-scale compared to small-scale screens. Moreover, action recognition performance in the visual periphery as probed by life-size human stick figures (see example in Figure 3) is different from action recognition performance probed by small point-light humans. For example, we have previously shown that action recognition of life-size stick figures (∼32° visual angle [VA]) is excellent up to 75° eccentricity (Fademrecht, Bülthoff, & de la Rosa, 2016, 2017). In contrast, the detection and discrimination of small point-light walkers (11° VA) was significantly decreased already at 12° eccentricities (Ikeda, Blake, & Watanabe, 2005; Ikeda, Watanabe, & Cavanagh, 2013; Thompson, Hansen, Hess, & Troje, 2007). These discrepant findings indicate that one cannot necessarily generalize the findings from experiments using desktop computers to more natural viewing conditions. For this reason, the current study examined action recognition using life-size action stimuli.

Size is not the only important factor to make viewing conditions appear natural. Humans are social beings that often gather together. As a result, actions are rarely viewed in isolation in real life. The presence of other people sufficiently close to a target actor could in principle induce well-known crowding effects. Previous research indicates that the deleterious effect of crowding on visual recognition of objects and actions is particularly pronounced in the visual periphery (Levi, 2008; Whitney & Levi, 2011). This is typically explained by the decline of visual acuity towards the periphery (Levi, 2008). Although crowding effects have also been reported for action recognition (Ikeda et al., 2013; Ikeda & Watanabe, 2016), little is known about whether crowding affects foveal and peripheral action recognition alike. There is some evidence that crowding already occurs for direction discrimination of point-light walkers in the fovea. Thornton and Vuong (2004) demonstrated that flankers’ walking direction influenced the perception of the walking direction of a target. Specifically, they found longer reaction times for reporting the walking direction of a target walker when target and flanking walkers faced different directions compared to when they faced the same direction. This effect of crowding on walking direction discrimination has also been found in the near periphery. Ikeda et al. (2013) showed that crowding occurred only with walking flankers, but not with scrambled walker flankers. These results suggest that crowding of biological motion is not due to low-level motion crowding effects but rather occurs on some higher level of visual processing, and that the presence of bystanders takes a toll on action recognition both in the fovea and near periphery.

Despite the clear demonstrations of crowding that these studies have provided, the degree to which these results apply to the ability to recognize actions when life-size stick figures are used is not known. Note that the recognition of certain types of actions (i.e., judging whether an action is a slap or a waving) is particularly relevant for social interactions in everyday life. For example, the ability to discriminate whether a person is waving or preparing to slap allows the observer to choose an appropriate action. There is some evidence that the discrimination of an action and its direction are partly dissociable suggesting that they are not mediated by the same mechanism (de la Rosa, Ekramnia, & Bülthoff, 2016; Ikeda & Watanabe, 2016). Hence, the degree to which action direction discrimination generalize to action discrimination is not known and therefore understanding the effect of crowding on action discrimination requires further investigation.

To this end, we conducted three experiments to examine action discrimination in the presence of other people under naturalistic conditions. In particular, we investigated whether the effect of crowding depends on target eccentricity and whether different types of crowd influence action discrimination differently. We used a setup in which actions were carried out by life-size human stick figures to provide both form and motion information of an action. Furthermore, our display allowed the assessment of action discrimination across the entire horizontal visual field (for more details, see Fademrecht et al., 2016, 2017).

## Experiment 1

We used a visual adaptation paradigm to examine action recognition. Visual adaptation refers to the transient change in percept of a stimulus after prolonged exposure to an adapting stimulus. For example, in colour adaptation, one perceives a white square to have a greenish tint after adapting to a red square. In an action adaptation paradigm, one of two actions (e.g., hug and clap) is typically used as an adaptor. Its presentation is followed by an ambiguous action which is a weighted average of the hug and clap actions. Participants frequently report that this perceptually ambiguous stimulus looks more like a clap after participants have adapted to a hug and vice versa (e.g., de la Rosa, Streuber, Giese, Bülthoff, & Curio, 2014; de la Rosa et al., 2016). Action adaptation effects can be well described in terms of neural populations (Webster, 2011) tuned to different actions (visual action channels). Adaptation causes the response of the visual action channel that is sensitive to the adapted action (e.g., hug) to be reduced. Hence during the subsequent presentation of the ambiguous stimulus, which contains originally an equal perceptual amount of hug and clap action, the hug channel’s response is smaller compared to that of the clap channel. Therefore, the observer perceives the ambiguous stimulus to look more like a clap. Interestingly, action adaptation and, more generally, visual adaptation effects agree well with physiological and brain imaging observations (Barraclough, Keith, Xiao, Oram, & Perrett, 2009; Barraclough & Jellema, 2011; Grill-spector & Malach, 2001; Kourtzi & Kanwisher, 2001). Because adaptation allows the selective targeting of neural populations, visual adaptation has also been termed the psychologist’s microelectrode (Frisby, 1979). Here, we used this method to selectively target neural processes underlying action recognition and to investigate their sensitivity to visual eccentricity and crowding.

In Experiment 1, we investigated the impact of two factors on the recognition of actions (hug and clap) performed by a life-size moving figure. These factors were the presence or absence of a nearby crowd and foveal or peripheral viewing conditions. We used an action adaptation paradigm and presented the test actions in central vision and at 40° eccentricity in three ‘crowd’ conditions: the moving figure was presented (a) *alone*, (b) in a crowd of *static* actors and (c) in a crowd of actors performing *idle* movements.

### Methods

#### Participants

We recruited 14 participants (8 females) from the local community of Tübingen. Participants received monetary compensation for their participation in the experiment. Their age ranged from 21 to 36 years (*M* = 25.5; *SD* = 4.1). Participants’ visual acuity was normal or corrected to normal with contact lenses (glasses could obliterate parts of the visual periphery). Participants in all experiments provided written informed consent prior to the experiment. The study was conducted in accordance with the Declaration of Helsinki and under the guidelines of the ethics board of the University of Tübingen.

#### Stimuli

We created two kinds of action stimuli: adaptor stimuli and test stimuli. In addition, we also generated crowd stimuli. All stimuli depicted moving human stick figures. Adaptor and test actions were carried out by one stick figure (target) presented at two positions on the screen (at fixation or at 40° eccentricity to the right). All stick figures were oriented so that the action was executed towards the participant.

#### Adaptor stimuli

In the current study, we chose two actions (clap and hug) to extend the adaptation paradigm designed by de la Rosa et al. (2014) to other social actions. The clap and hug actions were recorded from one actor via motion capture using a MVN Suit (XSens, Netherlands) containing 17 inertial and magnetic sensor modules. The sampling rate of the sensors was 120 Hz. Both actions started with a neutral body position and lasted 1,385 ms. Each action sequence ended at the point in time just before the actor started moving back to the neutral position. To display the actions, we mapped the recorded motion capture data onto a life-size avatar built as a grey stick figure (height: 170 cm, height 24° VA) using the Unity 3D (Unity Technologies, USA) game engine (see Figure 3). We used a stick figure instead of a more realistic avatar to prevent other visual cues like appearance or gender from influencing participants’ decisions about the action. Its position was defined by the position of a point midway between both hips.

#### Test stimuli

We used the same stick figures to create our test stimuli. These stimuli performed actions obtained by morphing between both adaptor stimuli (hug and clap) as described by de la Rosa et al. (2016) and Ferstl, Bülthoff, & de la Rosa (2017). First, we calculated weighted averages (weight_hug_ = 1 − weight_clap_) for each rotation of each body joint (e.g., knee, elbow) of the hug and the clap actions for each time-normalized frame (to assure that the two action movies that are being morphed have the same length). The morph weights ranged from 0.0 to 1.0 in steps of 0.1 to create nine morphed actions and two nonmorphed actions. All sequences lasted 1,385 ms. We presented these 11 actions at fixation to all participants and asked each of them to indicate verbally which action looked most ambiguous in terms of hug or clap. Which morph step was perceived as most ambiguous was individual to each participant and was used thereafter to create test stimuli specifically for each participant. We determined the test stimuli for each participant separately for the following reason. Measuring an adaptation aftereffect involves measuring the shift in perception of a stimulus after being adapted. This shift in perception is usually the largest around the point of subjective equality (ambiguity). By tailoring the ambiguous stimuli to each participant’s individual perception, we ensured that the shift in perception after adaptation was at the point of subjective equality and therefore maximal for each participant.

Once the most ambiguous action for each participants had been determined, the second step consisted of creating four additional morphed actions whose morph levels were equally spaced and symmetrical (step size: 0.025) around the chosen ambiguous action morph level for a total of five ambiguous test actions. For all participants, the weights we used varied between a minimum of 0.33 and a maximum of 0.63. We used five test actions that were perceptually discriminable by the participants so that they were not confronted with the exact same single test stimulus in all trials in the experiments reported later in this article.

#### Crowd stimuli

**Figure 1.**
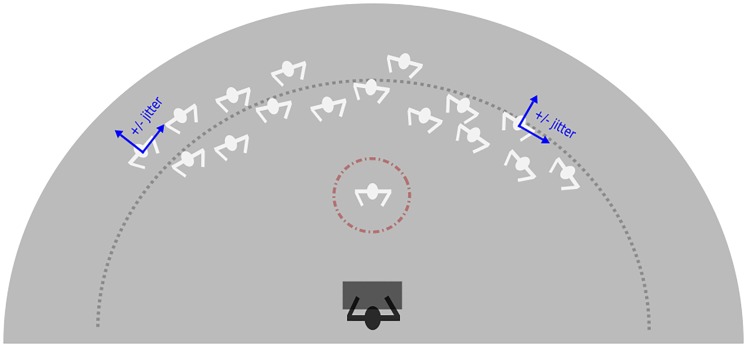
**Illustration of the spatial distribution of a crowd**. Participant (black) seated at a table in the middle of the panoramic screen arena. Red circle: Adaptor or test stimulus in front of the crowd. Dotted line (grey) illustrates the arc of a circle positioned at 6 m virtual distance away from the participant. The small blue axes indicate the jitter that was applied to each crowd member along the *x*- and *z*-axis along the arc of a circle. Displayed distances and angles are approximate and not to scale.

The crowd consisted of 16 stick figures. First, the stick figures were spatially distributed evenly (separated by 9.33° VA) along an arc of a circle positioned at 6 m virtual distance away from the participant (i.e., 2 m behind the *target,* who was acting out the adaptor and test actions). Thereby, the crowd spanned 140° VA. We then applied a random positional jitter along the *x* (left-right) and *z* (forward-backward) dimensions, which was maximally one eighth of the angular separation between adjacent crowd members (i.e., jitter range ±1.17° VA). This was meant to ensure the crowd’s spatial distribution appeared more natural (Figure 1). We created two different crowds: an idly moving crowd and a static crowd. All avatars of the idle crowd performed distinct small movements like stepping from one foot to the other or shaking one leg. They were animated for the same duration as the adaptor and test action stimuli. In the static crowd condition, we presented the first frame of each avatar animation for the same duration. The idle movements were selected from Rocketbox Libraries (Microsoft, Ireland) and applied to each figure of the crowd randomly. Selection criteria for the idle animations were that the stick figures never lifted their arms above the chest and that the animation was calm and moderately paced. We applied these criteria to ensure a clear distinction between the actions of the target stick figures and of those of the crowd members.

The most central crowd members were positioned at a horizontal distance of 4.7° VA ±1.17° from the target stick figure. The shoulder width of the target stick figure was 6° VA and varied between 3.6° VA and 5° VA for the crowd stick figures (due to spatial jitter along the *z* dimension). When the jitter in *x*- and *z*-coordinates was maximal, the distance between the shoulders of the crowd avatars and the target stick figure was 0.1° VA. When stick figures executed actions or idle movements, however, their arms were moving, this could lead to slight transient overlaps between them.

Target and crowd stick figures were clearly distinguishable, as the target stick figure was always presented in front of the crowd members (ca. 2 m).

#### Apparatus

**Figure 2.**
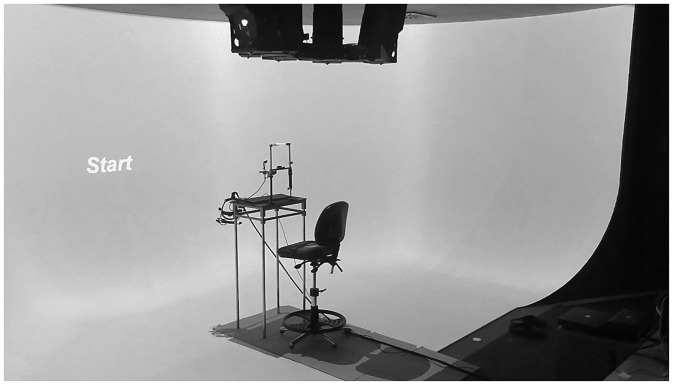
Experimental setup. Partial view of the semicylindrical screen with the participant chair positioned in its centre. The screen arena extends all the way to the participant chair.

We used a large panoramic screen with a semicylindrical two-dimensional (2D) projection system for the presentation of the stimuli (Figure 2; for more detailed information see Fademrecht et al., 2016). The screen was 3.2 m high and 7 m long. Its main vertical portion used for presenting the stimuli was 2.5 m away from the participants and covered 230° horizontally and 125° vertically of their visual field. It extended onto the floor towards the location where participants were seated (see Figure 2). The screen was equally lit with a mid-grey light. The Unity 3D (Unity Technologies) game engine in combination with a custom written control script was used to control presentation of the stimuli on the screen and collect keypress responses given by the participants on a keyboard. The use of the game engine in combination with the large screen leads to the impression of the avatars standing in a virtual room extending the real room in which the participants were sitting. The game engine ensured that all depth cues including correct lighting, occlusion and size scaling were present except for stereo cues. In this fashion, participants perceived the stick figure as being life size and located 4 m (target) or 6 m (crowd) away from the participants. During the experiment, participants were required to focus their gaze on a white fixation cross presented on the screen straight ahead of them.

**Figure 3.**
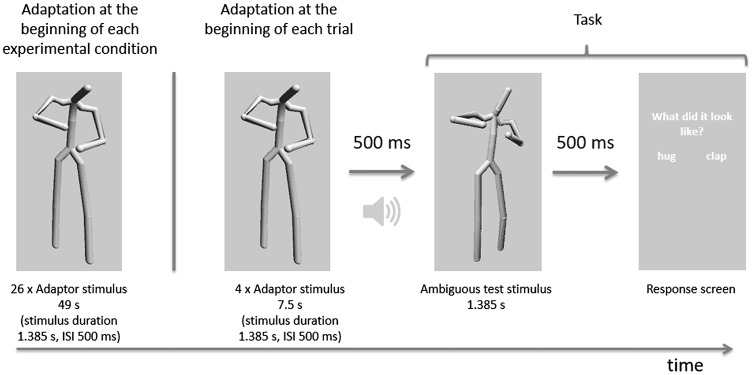
Adaptation paradigm: Timeline of the adaptation phase followed by an experimental trial of the experimental phase.

### Procedure and Design

Participants were seated in the middle of the screen arena and their heads were stabilized with a chin and forehead rest placed on a desk in front of them (see Figure 2). All tests were performed with the target presented at fixation and at 40° eccentricity to the right. All participants, before starting the tests described later in this article, had performed the verbal rating task mentioned earlier to design their personal ambiguous stimulus set.

### Baseline Condition

First, in a baseline condition, we probed each participant for his or her perception of the (ambiguous) test stimuli obtained earlier without the presentation of an adaptor. Each trial began with the presentation of a fixation cross and, after 500 ms, the test stimulus. The fixation cross was continuously present during stimulus presentation. After a blank of 500 ms the question: ‘What did it look like’? and the response options ‘hug’ and ‘clap’ appeared on the screen. Participants were asked to respond by pressing the appropriate key on a keyboard (keys 0 and 1 were used). The answer was not timed and there was no time restriction. Each of the five ambiguous test stimuli was presented three times in pseudorandom order. Presentation location (0° vs. 40° eccentricity) was also randomized.

### Adaptor Conditions

After the baseline measurements, we used an adaptation paradigm to test how sensitive action recognition is to our experimental manipulations. Our adaptation paradigm consisted of the presentation of an adaptor followed by a test stimulus. The original hug and clap actions served as adaptor stimuli whilst the five ambiguous morphs were used as test stimuli. We used this adaptation paradigm with all possible combinations of our experimental conditions: three crowd conditions (no crowd, static crowd and idly moving crowd), two different eccentricities (0° and 40° eccentricity) and two adaptor stimuli conditions (clap and hug). In the 0° eccentricity condition, adaptor and test stimuli were presented at 0°, whilst in the 40° eccentricity condition, adaptor and test were both presented at 40° eccentricity. The position of the crowd remained the same in both eccentricity conditions. In the no crowd condition, adaptor and test stimuli were presented alone on the screen. In the other two conditions, the crowd was visible during both the adaptation and test phases. We completely crossed all levels of the factors adaptor, eccentricity and crowd (2 × 2 × 3) and all participants were tested on every combination. Each factor combination was presented in a blocked fashion.

A schematic outline of an experimental block is shown in Figure 3. Each started with an initial adaptation phase followed by an experimental phase. During the initial adaptation phase, an adaptor stimulus (hug or clap) was shown 26 times (interstimulus interval [ISI] = 500 ms). This phase was included to maximize any adaptation aftereffect. Thereafter, the experimental phase consisted of several experimental trials. Each experimental trial started first with four presentations of the adaptor (ISI = 500 ms) to ‘top-up’ the initial adaptation. Immediately after that, a 500-ms blank screen was presented together with a 1000 Hz beep warning about the imminent display of the test stimulus. Subsequently, one of the five ambiguous test stimuli appeared followed by the answer screen (Figure 3). Participants had unlimited time to respond. The next experimental trial started immediately after the participants gave their response via keypress. Participants were asked to report their subjective feeling regarding the action category (hug or clap) of the test stimulus. Participants were explicitly instructed to judge the test stimuli, (not the adaptor), as either hug or clap. Within each block, each of the five ambiguous stimuli was presented three times for a total of 15 trials per block, whilst stimulus presentation was randomized.

An Eyelink II eye tracker mounted on the chin rest recorded participants’ eye movements. Participants were asked to fixate on the fixation cross during each trial. We had planned to remove from analysis trials for which participants moved their gaze away from the fixation cross by more than 2° during the stimulus presentation. Due to a technical error, however, the eye-tracking data could not be used. However, previous research using the same testing environment had shown that participants could reliably fixate (proportion of invalid trials was less than 0.8%) even during stimulation of the visual periphery (Fademrecht et al., 2016).

### Results

To analyse the data, we calculated the proportion of clap responses for each of the experimental conditions. Note that the results would be identical if we had chosen to calculate the proportion of hug responses instead of clap responses.

**Figure 4.**
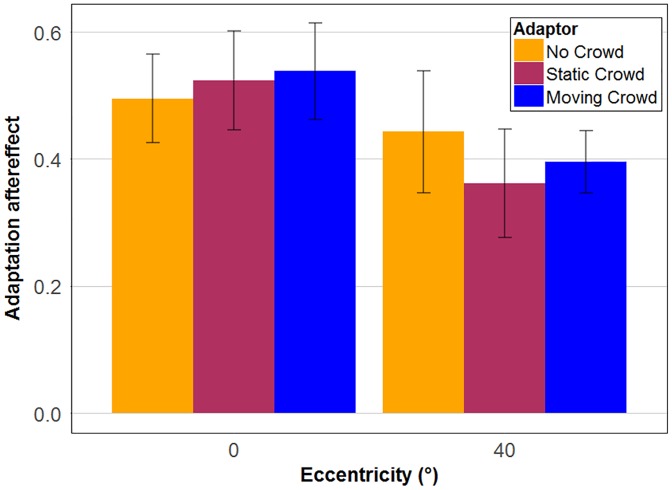
Overall adaptation aftereffects for each crowd condition (no crowd, static crowd and idly moving crowd) at 0° and 40° eccentricity. Colours represent the three crowd conditions. Error bars represent standard errors of the mean.

Our main goal in this experiment was to assess the adaptation aftereffect (defined here as the difference of proportion of clap responses between hug and clap adaptor conditions) on action perception (see Figure 4). A two-way repeated measure analysis of variance (ANOVA) with crowd and eccentricity as within-subject factor demonstrated that the main effects of crowd, *F*(1, 13) = 1.57; η^2^_partial_ = 0.11; *p* = .232, and eccentricity, *F*(2, 26) = 0.16; η^2^_partial_ = 0.01; *p* = .853, as well as their interaction, *F*(2, 26) = 0.81; η^2^_partial_ = 0.06; *p* = .457, were all nonsignificant. Hence, there was no difference in adaptation aftereffects in central and peripheral vision and the static crowd as well as the idly moving crowd had little influence on the adaptation aftereffect in comparison to the no crowd condition. Moreover, all adaptation aftereffects were significantly different from zero (Holm corrected) in all crowd conditions at 0° and 40° eccentricity (all *p* < .001).

### Discussion

Experiment 1 revealed that neither the presence of a crowd nor peripheral presentation significantly affected action adaptation. Similar adaptation aftereffects in central vision and at 40° eccentricity indicate that action recognition mechanisms that are susceptible to adaptation can discriminate actions even in the far periphery. These results are in line with other research that suggests that participants have little difficulty in recognizing moving actions in the visual periphery up to 45° eccentricity (Fademrecht et al., 2016).

The presence of a static or an idly moving crowd did not influence the effects of action adaptation. This finding is in accordance with previous research which showed that low-level adaptation aftereffects (e.g., orientation adaptation aftereffects) are not at all or little affected by crowding. For example, Blake, Tadin, Sobel, Raissian, and Chong (2006) demonstrated that crowding does not reduce the orientation adaptation aftereffect, at least when high contrast stimuli are presented. Similarly, Pelli and Tillman (2008) reported that crowding affects the discrimination of target orientation but has little effect on the occurrence of an orientation adaptation aftereffect. Hence, one explanation might be that adaptation aftereffects, in general, are little affected by crowding.

An alternative explanation could be that neural populations (action channels) are sensitive to a specific action (akin to the action-sensitive units in the Giese and Poggio model [2003]). According to this view, in order for the crowd to induce crowding effects, the crowd actions need to activate at least one of the two action channels (clap or hug channel) involved in the perception of the test stimulus. Yet, neither the static nor the idle crowd showed actions that could activate those channels. Hence, adaptation aftereffects should be unaffected by these crowds. According to this explanation, a crowd might only modify the adaptation aftereffect if its members display clap and hug actions. In Experiment 2, we tested this hypothesis.

## Experiment 2

Experiment 2 replicated Experiment 1 with the only difference that we used a crowd whose members were carrying out the adaptor actions (active crowd). We examined action adaptation aftereffects with the same adaptation paradigm whilst manipulating eccentricity (0° vs. 40°) and crowd (no crowd vs. active crowd).

### Methods

#### Participants

We recruited 16 participants (10 females) from the local community of Tübingen. Their age ranged from 21 to 56 years (*M* = 31.06, *SD* = 9.64).

#### Stimuli

The adapting stimuli used were the same as for Experiment 1; similarly, the test stimuli were generated separately for each participant.

#### Crowd stimuli

For this experiment, we created a crowd as described in Experiment 1, except that each member of the crowd was animated randomly by either a hug or a clap action (i.e., 50% of the crowd members were performing the hug action and 50% performed the clap action), we refer to this crowd as the *active crowd*.

### Design and Procedure

There were only two different crowd conditions: no crowd and active crowd; all other details of the design and the procedure of Experiment 1 were repeated.

### Results

**Figure 5.**
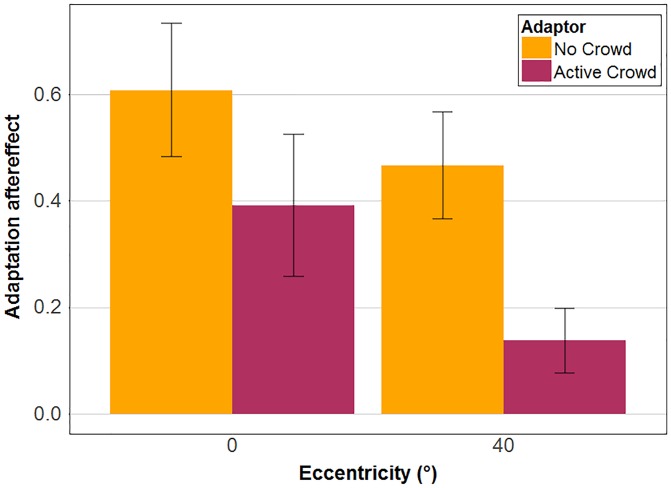
Adaptation aftereffects for both crowd conditions at 0° and 40° eccentricity. Colours represent the two crowd conditions. Error bars represent standard errors of the mean.

In order to assess the effects of the crowd and eccentricity conditions on the adaptation aftereffect (see Figure 5), a two-way repeated measure ANOVA with crowd and eccentricity as within-subject factors was calculated. The results show that the adaptation aftereffect was significantly stronger for adaptation at 0° eccentricity than at 40°, *F*(1, 15) = 4.64, *p* = .048, η^2^_partial_ = 0.24. Further, the crowd had a significant main effect, *F*(1, 15) = 13.14, *p* < .01, η^2^_partial_ = 0.47; the active crowd significantly reduced the adaptation aftereffect in comparison to the no crowd condition. The interaction between eccentricity and crowd factors was nonsignificant, *F*(1, 15) = 0.73, *p* = .407, η^2^_partial_ = 0.05. In addition, we found all adaptation aftereffects to be significantly different from zero (all *p* < .05; Holm corrected).

### Discussion

We found that the active crowd significantly decreased the action adaptation aftereffect in central and peripheral vision. This is in line with the hypothesis that the effect of the crowd is due to crowd actions activating the same neural populations (action channels) as the adaptor and test stimuli. Hence, we suggest that crowding effects are observed in our adaptation paradigm when adaptor, test and crowd actions are sufficiently similar to each other.

Crowding effects might be task specific. For example, it has been previously reported that crowding affects performance in adaptation but not detection tasks (Aghdaee, 2005; Blake et al., 2006; He, Cavanagh, & Intriligator, 1996; Pelli, Palomares, & Majaj, 2004; Pelli & Tillman, 2008). This suggests that crowded environments might affect performance in adaptation and recognition tasks differently. We wanted to assess whether our evidence of a crowding effect could also be found using an action recognition task without adaptation. In Experiment 3, we tested participants’ performance in an action recognition task whilst again manipulating crowd and eccentricity.

## Experiment 3

### Methods

#### Stimuli

The original clap and hug actions described in Experiment 1 were used. We used no morphed actions in this experiment.

#### Crowd stimuli

The same crowd stimuli as in Experiment 2 were used.

#### Participants

We recruited 14 participants (eight females) from the local community of Tübingen. Their age ranged from 19 to 35 years (*M* = 26.08, *SD* = 4.7).

### Design and Procedure

Prior to the experiment, participants were instructed verbally to categorize the action of the target actor as either clap or hug. Participants were seated in the middle of the panoramic screen with their head resting on a chinrest. Whilst participants were fixating a fixation cross in the middle of the screen, the clap and hug actions were presented at 0° and 40° eccentricity randomly in two crowd conditions (no crowd or active crowd). Both actions were shown 20 times at each eccentricity (0° and 40°), which resulted in 160 trials (2 Eccentricities × 2 Crowd Conditions × 2 Actions × 20 Repetitions). After stimulus onset, participants indicated their response via keypress and were instructed to respond as fast and accurately as possible, but response time was unlimited. We recorded participants’ accuracy and reaction times as measures of recognition performance. An Eyelink II eye tracker mounted on the chin rest was used to control participants’ fixation. Participants were asked to direct their gaze onto the fixation cross and were told that trials where they looked to the side could not be used for analysis.

### Results

We removed from the analysis trials for which participants moved their gaze away from the fixation cross by more than 2° during the stimulus presentation. We assessed recognition performance in terms of reaction times and accuracy. The results for these two dependent variables are presented separately, whilst we considered only reaction times for correct responses.

**Figure 6.**
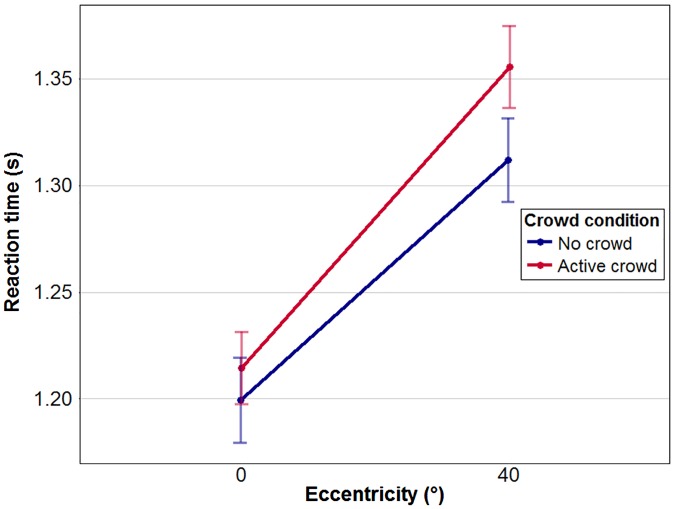
Mean reaction times for each crowd condition at 0° and 40° eccentricity. Colours represent the two crowd conditions. Error bars represent standard errors of the mean.

Visual inspection of Figure 6 shows that reaction times were quicker in central vision than at 40° eccentricity for both crowd conditions. A two-way repeated measure ANOVA with crowd and eccentricity as within-subject factors confirmed the visual impression. Reaction times in the periphery were significantly slower—main effect of eccentricity, *F*(1, 13) = 59.88, *p* < .001, η^2^_partial_ = 0.83. The presence of the crowd did not have any significant effect, *F*(1, 13) = 0.14; η^2^_partial_ = 0.01; *p* = .715, and the interaction between crowd condition and eccentricity was also not significant, *F*(1, 13) = 0.1.28; η^2^_partial_ = 0.10; *p* = .281.

**Figure 7.**
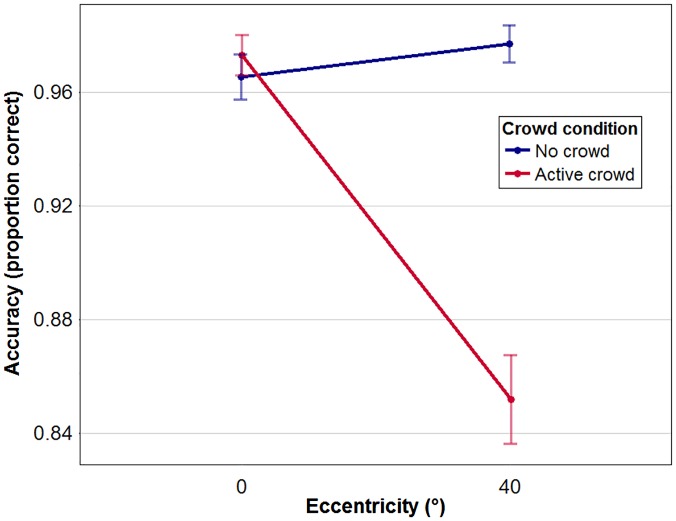
Mean proportion correct for each crowd condition at 0° and 40° eccentricity. Colours represent the two crowd conditions. Error bars represent standard errors of the mean. Note the enlarged scale of the ordinate.

The mean accuracy for all conditions was well above chance level (see Figure 7). We analysed the accuracy results using a two-way repeated measure ANOVA with crowd and eccentricity as within-subject factors. Participants were significantly more accurate when there was no crowd than when the active crowd was present—main effect of crowd, *F*(1, 13) = 5.49, *p* = .037, η^2^_partial_ = 0.31. There was no main effect of eccentricity, *F*(1, 13) = 4.18, *p* = .063, η^2^_partial_ = 0.26. The influence of the crowd on the proportion correct significantly depended upon target eccentricity—interaction between crowd and eccentricity, *F*(1, 13) = 8.64, *p* = .012, η^2^_partial_ = 0.42—the presence of the crowd reduced accuracy for a target viewed in the periphery more than at fixation (see Figure 7).

### Discussion

Our results suggest that both the presence of a crowd and the location of the target in the visual space of the observer have an effect on action recognition. Participants were slower to respond when a target was presented in their visual periphery, and they were also less accurate at that position when the target was surrounded by an active crowd. These results corroborate what we have observed in Experiment 2 with an adaptation paradigm. Moreover, these findings also agree with a previous study that had showed that action recognition is possible in far periphery up to 60° but tends to decrease for eccentricities larger than 45° (Fademrecht et al., 2016). Hence, these results suggest that the presence of a crowd reduces the accuracy of action recognition in the periphery if the crowd is executing similar actions to that of the target actor, but this effect is not observed in central vision.

## General Discussion

### On the Origin of Crowding Effects

The simple presence of a crowd was not enough to modulate the action adaptation aftereffect in our adaptation experiments, even when it was animated with idle movements. Rather, a crowding effect, demonstrated by a reduced adaptation aftereffect, was only observed when the crowd exhibited the same actions as the adaptor stimuli (hug or clap). Hence, our results suggest that bystanders carrying out actions similar to the target person interfere with the observer’s ability to categorize actions.

Previous research has shown that other adaptation effects (e.g., orientation adaptation, motion adaptation) are little affected by crowding (Aghdaee, 2005; He et al., 1996; Pelli et al., 2004; Whitney & Levi, 2011). For example, Blake et al. (2006) showed that crowding does not reduce the adaptation aftereffect for simple features (e.g., orientation) when stimuli are presented with high contrast. Similarly, Pelli and Tillman (2008) reported that crowding prevents the ability to judge target orientation whilst not affecting the occurrence of orientation-specific adaptation aftereffects. Whilst these studies report that orientation adaptation is little affected by crowding, the suggested underlying mechanism might not apply to the results of our study. Specifically, Pelli and Tillman explain these effects within a two-step object recognition process. In the first step, which is susceptible to adaptation, object features are detected. In the second step, features are combined. According to Pelli and Tillman, feature combination is susceptible to crowding. This explanation is difficult to reconcile with the results of our study because the visual features critical for the recognition of actions are assumed to be combinations of ‘object’ features of Pelli and Tillman’s first stage (Pelli & Tillman, 2008). Hence, according to Pelli and Tillman's explanation, one would expect action adaptation to be affected by crowding, which is not what we found when testing with static and idle crowds.

Ikeda et al. (2013) argued that crowding of biological motion stimuli is a high-level effect. Biological motion flankers influenced the recognition of the walking direction of a target stimulus, whereas scrambled biological motion flankers had no influence. In line with the idea of a high-level crowding effect, we found in the current study that neither a static nor an idle crowd (i.e., which performed other movements than the target stick figure) reduced action adaptation aftereffects. This suggests that low-level shape and motion cues are not effective to illicit action adaptation crowding effects. Instead, we found reduced action adaptation aftereffects only when the crowd actors performed the same actions as the target actor.

Are the differences between the adaptation effects in the idle and active crowd simply owed to the higher amount of motion in the active crowd condition? In order to assess this possibility, we quantified the amount of motion of the idle and active crowd. To that end, we created binarized movies of each crowd. In each frame, all pixels belonging to the stick figure had a value of 1 and the pixels belonging to the background had a value of 0. We then calculated how many pixels changed their values from 1 to 0 or the other way around between two successive movie frames. This measure, averaged across all crowd members, represents the amount of motion visible in the image plane calculated across two movie frames. We have shown previously that this measure of motion correlates with the 2D motion energy of the stimuli (Fademrecht et al., 2017). The analysis shows that the amount of movement is indeed significantly higher in the active crowd than in the idle crowd, *t*(100.59) = 13.91, *p* < .001, across the movie frames.

Yet, we do not think that the difference in adaptation effects between active and idle crowd is primarily driven by these differences in motion. First, the motion of the idle crowd was clearly visible and participants verbally reported that they easily perceived the idle movements even in the periphery. More importantly, the increase in visible motion from the static crowd (0 pixel changed between two successive trials) to the idle crowd (3.09 pixels changed on average between successive trials) was comparable to the increase in motion from the idle crowd to the active crowd (7.66 pixel changed on average between two successive trials). If differences in adaptation effect were solely bound to these differences between crowds, we would expect to also find an adaptation difference between the idle and the static crowd condition. Yet, our results showed no such difference, suggesting that the amount of motion alone cannot explain the observed differences in adaptation aftereffects. Second, unpublished data from our lab show that local motion is unable to induce action adaptation effects. Specifically, we remapped arm motions onto the legs and vice versa thereby distorting the overall action percept but retaining local joint angles that have been shown to be important for action recognition. These remapped actions did not induce any adaptation effect. Therefore, we do not expect motion alone to affect action adaptation. In sum, we argue that differences in adaptation aftereffects between crowd conditions are not a mere reflection of motion differences between crowds. Rather, our findings imply that crowding effect on the recognition of human action stimuli is a high-level effect, possibly at the level at which individual actions are represented, as suggested by Ikeda et al. (2013).

### A Perceptual Channel Model for Action Adaptation Crowding Effects

How could these results be understood in terms of a perceptual channel model of action recognition? Action-sensitive neural units are often tuned to a particular feature of an action (e.g., Perrett et al., 1989) and computational models of action recognition explain how these tuning characteristics can be derived within the visual processing hierarchy (Giese & Poggio, 2003). In low-level vision, the response properties of neural units have been described in terms of perceptual channels, which are tuned to a particular property of the stimulus (e.g., orientation, spatial frequency) and have a limited bandwidth (Campbell & Robson, 1968). We suggest to describe action recognition effects in terms of action-sensitive channels. This approach is not uncommon as in psychophysics neural processes are often modelled as perceptual channels which relate the response of the process to a physical property of the stimulus (Benton, Thirkettle, & Scott-Samuel, 2016; Bower, Bian, & Andersen, 2012; Gardner, 1973; To, Lovell, Troscianko, & Tolhurst, 2010; Webster, 2012). These perceptual channel models assume several channels each tuned to a particular stimulus property (Webster, 2011). During recognition, the responses of these perceptual channels are pooled (Riesenhuber and Poggio, 1999a, 1999b, 2000).

As proposed in Experiment 1, one can envision that in our experiments two action-sensitive channels are at work; one is mainly tuned to a hug and the other to a clap. The repeated presentation of a hug during adaptation reduces strongly the response of the hug-sensitive channel. When participants are subsequently presented with an ambiguous stimulus in the test phase, the clap-sensitive channel responds more strongly than the hug channel. Consequently, participants perceive the ambiguous action to look more like a clap and an adaptation aftereffect can be measured. Note that under this conceptualization, we make no assumptions about *how* adaptation reduces the action channel response; different neural mechanisms, such as changes in gain, in selectivity, and response timing (Grill-Spector, Henson, & Martin, 2006), have been proposed to explain the reductions of response intensity at the neural population level.

Why does only the active crowd reduce the adaptation aftereffect? In the active crowd condition, the crowd members performed both clap and hug actions during adaptation. As in the other crowd conditions, the hug adaptor induces a strong reduction of the hug channel response. This response, though, cannot be further reduced by the hugging members of the active crowd, whilst, in contrast, the response of the clap channel is reduced by the crowd members executing a clap. Therefore, the presence of the active crowd results in a smaller response difference between hug and clap channels compared to the other crowd conditions and the aftereffect is correspondingly smaller.

In Experiment 2, we found that the presence of the active crowd modulated the adaptation aftereffect in central vision as well as in the visual periphery. In Experiment 3, where we used a recognition task instead of an adaptation paradigm, the active crowd only influenced peripheral vision. The difference in results between Experiments 2 and 3 is in line with the reports of Pelli and Tillman (2008) and Blake et al. (2006) that suggest that adaptation paradigms and recognition tasks probe different stages of visual processes.

### On the Presentation Style of the Crowd

Why did we choose to present a crowd during both the adaptation phase and the test phase? If we had presented a crowd only during the test phase, its sudden appearance at test might have caused a shift of attention towards the additional actors, but this would have occurred in the crowd condition only. Avoiding this surprise effect thus eliminates a possible confound in the interpretation of the results.

### Increased Ecological Validity

An important aim of this study was to investigate action recognition whilst using a paradigm with increased ecological validity compared to previous research in this field. To this end, we chose as action stimuli life-size human stick figures that performed hug and clap actions. Although the resemblance between stick figures and real-life actors may be disputed, we argue here that using simplified stick figures was advantageous: We minimized the chances that our results were clothing-, body shape-, or gender-specific. Despite their simplicity, stick figures provide a clear step towards real-life actors when compared to the point-light walkers used in many previous studies. In addition, by the use of a panoramic display, we were able to test a large portion of the horizontal extent of the visual field. The visual periphery plays a role in everyday situations and by investigating peripheral action recognition we take a step towards increasing the ecological validity of our findings.

Our research is in line with previous efforts to examine social cognitive processes, such as action recognition, under more natural experimental conditions (De Jaegher et al., 2010; Schilbach et al., 2013). Previous work in our lab, for example, has shown that action recognition processes rely on different mechanisms depending on whether participants are passive observers of a social action or are actively engaged in it (de la Rosa, Frestl, & Bülthoff, 2016). Our research has shown that action recognition under these conditions is quite different from standard laboratory settings. Therefore, we suggest that probing action recognition under these conditions provides more valid insights into action recognition in real life.

## Conclusion

In conclusion, we find that the recognition of the action of a person can be influenced by the presence of a surrounding crowd. However, this crowd effect is dependent upon what the crowd is doing. Action perception is only affected when the crowd and the target person perform similar actions. Finally, the magnitude of this crowd effect on action perception is dependent upon the nature of the task employed and the position in visual space of the target action.

## References

[bibr1-2041669517743521] Aghdaee S. M. (2005) Adaptation to spiral motion in crowding condition. Perception 34: 155–162. doi:10.1068/p5298. 15832566 10.1068/p5298

[bibr2-2041669517743521] Barraclough N. E. Jellema T. (2011) Visual aftereffects for walking actions reveal underlying neural mechanisms for action recognition. Psychological Science 22: 87–94. doi:10.1177/0956797610391910. 21164175 10.1177/0956797610391910

[bibr3-2041669517743521] Barraclough N. E. Keith R. H. Xiao D. Oram M. W. Perrett D. I. (2009) Visual adaptation to goal-directed hand actions. Journal of Cognitive Neuroscience 21: 1806–1820. doi:10.1162/jocn.2008.21145. 18855549 10.1162/jocn.2008.21145

[bibr5-2041669517743521] Benton C. P. Thirkettle M. Scott-Samuel N. E. (2016) Biological movement and the encoding of its motion and orientation. Scientific Reports 6: 22393 doi:10.1038/srep22393. 26925870 10.1038/srep22393 PMC4772625

[bibr7-2041669517743521] Blake R. Tadin D. Sobel K. V. Raissian T. A. Chong S. C. (2006) Strength of early visual adaptation depends on visual awareness. Proceedings of the National Academy of Sciences of the United States of America 103: 4783–4788. doi:10.1073/pnas.0509634103. 16537384 10.1073/pnas.0509634103 PMC1400587

[bibr8-2041669517743521] Bower J. D. Bian Z. Andersen G. J. (2012) Effects of retinal eccentricity and acuity on global motion processing. Attention, Perception, & Psychophysics 74: 942–949. doi:10.3758/s13414-012-0283-2.Effects. 10.3758/s13414-012-0283-2 PMC3381858 22382583

[bibr9-2041669517743521] Campbell F. W. Robson J. G. (1968) Application of fourier analysis to the visibility of gratings. The Journal of Physiology 197: 551–566. doi: 10.1113/jphysiol.1968.sp008574. 5666169 10.1113/jphysiol.1968.sp008574 PMC1351748

[bibr14-2041669517743521] De Jaegher H. Di Paolo E. Gallagher S. (2010) Can social interaction constitute social cognition? Trends in Cognitive Sciences 14: 441–447. doi:10.1016/j.tics.2010.06.009. 20674467 10.1016/j.tics.2010.06.009

[bibr15-2041669517743521] de la Rosa S. Ekramnia M. Bülthoff H. H. (2016) Action recognition and movement direction discrimination tasks are associated with different adaptation patterns. Frontiers in Human Neuroscience 10: 56 doi:10.3389/fnhum.2016.00056. 26941633 10.3389/fnhum.2016.00056 PMC4763159

[bibr16-2041669517743521] de la Rosa S. Ferstl Y. Bülthoff H. H. (2016) Visual adaptation dominates bimodal visual-motor action adaptation. Scientific Reports 6: 23829 doi:10.1038/srep23829. 27029781 10.1038/srep23829 PMC4814912

[bibr17-2041669517743521] de la Rosa S. Streuber S. Giese M. A. Bülthoff H. H. Curio C. (2014) Putting actions in context: Visual action adaptation aftereffects are modulated by social contexts. PLoS ONE 9: e86502 doi:10.1371/journal.pone.0086502. 24466123 10.1371/journal.pone.0086502 PMC3899271

[bibr18-2041669517743521] Fademrecht L. Bülthoff I. de la Rosa S. (2016) Action recognition in the visual periphery. Journal of Vision 16: 1–14. doi:10.1167/16.3.33.doi. 10.1167/16.3.33 26913625

[bibr19-2041669517743521] Fademrecht L. Bülthoff I. de la Rosa S. (2017) Action recognition is viewpoint-dependent in the visual periphery. Vision Research 135: 10–15. doi:10.1016/j.visres.2017.01.011. 28435124 10.1016/j.visres.2017.01.011

[bibr20-2041669517743521] Ferstl Y. Bülthoff H. H. de la Rosa S. (2017) Action recognition is sensitive to the identity of the actor. Cognition 166: 201–206. doi:10.1016/j.cognition.2017.05.036. 28582683 10.1016/j.cognition.2017.05.036

[bibr21-2041669517743521] Frisby, J. P. (1979). *Seeing*. Oxford University Press. doi:10.1007/s007690000247.

[bibr22-2041669517743521] Gardner T. (1973) Evidence for independent in tachistoscopic parallel channels perception. Cognitive Psychology 155: 130–155. doi:/10.1016/0010-0285(73)90009-1.

[bibr24-2041669517743521] Giese M. A. Poggio T. (2003) Neural mechanisms for the recognition of biological movements. Nature Reviews Neuroscience 4: 179–192. doi:10.1038/nrn1057. 12612631 10.1038/nrn1057

[bibr25-2041669517743521] Grill-Spector K. Malach R. (2001) fMR-adaptation: A tool for studying the functional properties of human cortical neurons. Acta Psychologica 107: 293–321. doi:10.1016/S0001-6918(01)00019-1. 11388140 10.1016/s0001-6918(01)00019-1

[bibr26-2041669517743521] Grill-Spector K. Henson R. Martin A. (2006) Repetition and the brain: Neural models of stimulus-specific effects. Trends in Cognitive Sciences 10: 14–23. doi:10.1016/j.tics.2005.11.006. 16321563 10.1016/j.tics.2005.11.006

[bibr29-2041669517743521] He S. Cavanagh P. Intriligator J. (1996) Attentional resolution and the locus of visual awareness. Nature 383: 334–337. doi:10.1038/383334a0. 8848045 10.1038/383334a0

[bibr31-2041669517743521] Ikeda H. Blake R. Watanabe K. (2005) Eccentric perception of biological motion is unscalably poor. Vision Research 45: 1935–1943. doi:10.1016/j.visres.2005.02.001. 15820512 10.1016/j.visres.2005.02.001

[bibr32-2041669517743521] Ikeda H. Watanabe K. Cavanagh P. (2013) Crowding of biological motion stimuli. Journal of Vision 13: 1–6. doi:10.1167/13.4.20.doi. 10.1167/13.4.20 23532908

[bibr33-2041669517743521] Ikeda H. Watanabe K. (2016) Action congruency influences crowding when discriminating biological motion direction. Perception 45: 1046–1059. doi:10.1177/0301006616651952. 27251168 10.1177/0301006616651952

[bibr35-2041669517743521] Keefe B. D. Wincenciak J. Jellema T. Ward J. W. Barraclough N. E. (2016) Action adaptation during natural unfolding social scenes influences action recognition and inferences made about actor beliefs. Journal of Vision 16: 9 doi:10.1167/16.9.9. 10.1167/16.9.9 27472496

[bibr36-2041669517743521] Kourtzi Z. Kanwisher N. (2001) Representation of perceived object shape by the human lateral occipital cortex. Science 293: 1506–1509. doi:10.1126/science.1061133. 11520991 10.1126/science.1061133

[bibr38-2041669517743521] Levi D. M. (2008) Crowding – An essential bottleneck for object recognition: A mini-review. Vision Research 48: 635–654. doi:10.1016/j.visres.2007.12.009. 18226828 10.1016/j.visres.2007.12.009 PMC2268888

[bibr41-2041669517743521] Pelli D. G. Palomares M. Majaj N. J. (2004) Crowding is unlike ordinary masking: Distinguishing feature integration from detection. Journal of Vision 4: 12 doi:10.1167/4.12.12. 10.1167/4.12.12 15669917

[bibr42-2041669517743521] Pelli D. G. Tillman K. a. (2008) The uncrowded window of object recognition. Nature Neuroscience 11: 1129–1135. doi:10.1038/nn1208-1463b. 18828191 10.1038/nn.2187 PMC2772078

[bibr43-2041669517743521] Perrett D. I. Harries M. H. Bevan R. Thomas S. Benson P. J. Mistlin a. J. Ortega J. E. (1989) Frameworks of analysis for the neural representation of animate objects and actions. The Journal of Experimental Biology 146: 87–113. Retrieved from http://www.ncbi.nlm.nih.gov/pubmed/2689570. 2689570 10.1242/jeb.146.1.87

[bibr44-2041669517743521] Riesenhuber, M., & Poggio, T. (1999a). Are cortical models really bound by the “binding problem”?, *24*, *Neuron*, 87–93. doi:10.1016/S0896-6273(00)80824-7. 10.1016/s0896-6273(00)80824-7 10677029

[bibr45-2041669517743521] Riesenhuber M. Poggio T. (1999b) Hierarchical models of object recognition in cortex. Nature Neuroscience 2: 1019–1025. doi:10.1038/14819. 10526343 10.1038/14819

[bibr46-2041669517743521] Riesenhuber M. Poggio T. (2000) Models of object recognition. Nature Neuroscience 3 Suppl: 1199–204. doi:10.1038/81479. 11127838 10.1038/81479

[bibr47-2041669517743521] Schilbach L. Timmermans B. Reddy V. Costall A. Bente G. Schlicht T. Vogeley K. (2013) Toward a second-person neuroscience. The Behavioral and Brain Sciences 36: 393–414. doi:10.1017/S0140525X12000660. 23883742 10.1017/S0140525X12000660

[bibr49-2041669517743521] Thompson B. Hansen B. C. Hess R. F. Troje N. F. (2007) Peripheral vision: Good for biological motion, bad for signal noise segregation? Journal of Vision 7: 1–7. doi:10.1167/7.10.12.Introduction. 10.1167/7.10.12 17997681

[bibr50-2041669517743521] Thornton I. M. Vuong Q. C. (2004) Incidental processing of biological motion. Current Biology 14: 1084–1089. doi:10.1016/j.cub.2004.06.025. 15203001 10.1016/j.cub.2004.06.025

[bibr51-2041669517743521] To M. P. S. Lovell P. G. Troscianko T. Tolhurst D. J. (2010) Perception of suprathreshold naturalistic changes in colored natural images. Journal of Vision 10: 12 doi:10.1167/10.4.12. 10.1167/10.4.12 20465332

[bibr56-2041669517743521] Webster M. (2012) Evolving concepts of sensory adaptation. F1000 Biology Reports 4: 1–7. doi:10.3410/B4-21. 23189092 10.3410/B4-21 PMC3501690

[bibr57-2041669517743521] Webster M. A. (2011) Adaptation and visual coding. Journal of Vision 11: 1–23. doi:10.1167/11.5.3. 10.1167/11.5.3 PMC3245980 21602298

[bibr58-2041669517743521] Whitney D. Levi D. M. (2011) Visual crowding: A fundamental limit on conscious perception and object recognition. Trends in Cognitive Sciences 15: 160–168. doi:10.1016/j.tics.2011.02.005. 21420894 10.1016/j.tics.2011.02.005 PMC3070834

